# Overview of Available Functioning Data in Switzerland: Supporting the Use of Functioning as a Health Indicator Alongside Mortality and Morbidity

**DOI:** 10.3389/ijph.2024.1607366

**Published:** 2024-08-14

**Authors:** Beatriz Moreira, Jsabel Hodel, Melissa Selb, Jiin Kim, Carolina Fellinghauer, Jerome Bickenbach, Carla Sabariego

**Affiliations:** ^1^ Faculty of Health Sciences and Medicine, University of Lucerne, Lucerne, Switzerland; ^2^ Swiss Paraplegic Research, Nottwil, Switzerland; ^3^ ICF Research Branch, Nottwil, Switzerland; ^4^ Center for Rehabilitation in Global Health Systems, WHO Collaborating Centre, Lucerne, Switzerland

**Keywords:** functioning, disability, healthy ageing, population health, ICF linking rules

## Abstract

**Objectives:**

To identify official sources that routinely collect data on functioning in Switzerland, to provide an overview of the existing data and its comparability, and to assess the extent to which the data is suitable for developing a functioning metric and indicator.

**Methods:**

Data sources were identified through an iterative search. Standardized rules were applied to map the functioning information assessed by the sources using a current WHO functioning and disability survey as a reference framework for the content comparison.

**Results:**

Four sources were identified: the Swiss Survey of Health, Ageing and Retirement in Europe (SHARE), the Swiss Health Survey (SHS), the Lausanne cohort 65+ (Lc65+), and the Swiss Household Panel (SHP). All tools addressed sleep functions, energy level, emotional functions, and sensation of pain. Additionally, nine functioning categories were common across three sources.

**Conclusion:**

Population data sources in Switzerland routinely collect comparable functioning data, which can serve as the basis for creating a functioning indicator. Among others, this indicator is relevant to complement mortality and morbidity data and to support both the estimation of rehabilitation and long-term care needs.

## Introduction

The world’s population is rapidly ageing, creating unprecedent challenges for society and health systems. By 2050, the number of people aged 60 and above is projected to double to 2.1 billion [1]. Europe is at the forefront of this demographic transition, with more than 30% of its citizens expected to be 65 years of age or older by 2100 [2]. As life expectancy increases, so does the prevalence of non-communicable diseases (NCDs). Switzerland follows the trend, with the Swiss living longer and at the same time experiencing NCDs to a degree not seen in the past. By 2050, 30% of the population will likely be 65 or older [3] and about 50% of those will have at least one chronic illness [4]. The increasing number of people living with chronic conditions is driving a higher demand for healthcare services, especially rehabilitation and long-term care (LTC) [5, 6], putting pressure on resource-constrained systems.

According to the United Nations’ Decade of Healthy Ageing [1], health spending should not just extend life but also improve health and wellbeing. To achieve those goals, policymakers generally assess morbidity and mortality as key health indicators, based on the World Health Organization’s (WHO) International Classification of Diseases [7] and use these statistics for planning and setting priorities in health systems. But mortality and morbidity alone do not capture the lived experience of health [8], i.e., the extent to which health limitations and disability are present in a population, and the impact they have on people’s everyday life.

The concept of functioning operationalizes health from a biopsychosocial approach that fully acknowledges the context of a person or population. The definition of human functioning was introduced by WHO in its International Classification of Functioning, Disability and Health (ICF) [9]. Functioning is a universal experience and measured on a continuum from none to extreme problems. It consists of the components of *body functions and structures*, *activities* and *participation,* that is, the set of all functions and structures of the human body and all of the behaviors, actions, tasks that a person performs [9]. This understanding of functioning goes beyond the activities and instrumental activities of daily living (ADL and IADL), which are commonly used in gerontological research [10] to measure a person’s functional status. ADL and IADL are part of functioning and coded in the ICF within the *activities* and *participation* component. Functioning, however, includes more domains than the usual ADL and IADL, and goes beyond them for also including *body functions* and *structures* components. It considers further important aspects related to the overall functioning of a person or population, for example, body functions such as energy and drive functions, emotional functions, and sensation of pain which are known to have a high impact on functioning [11]. Moreover, functioning is understood as the result of the interaction between the health condition and the context of a person - *environmental* and *personal factors* [9]. The health condition and the context of a person are not part of functioning, but rather determinants of it. They are critical to understanding functioning because people with the same health condition may experience, depending on their context, very different levels of functioning in their daily lives.

Using a functioning indicator requires the construction of a functioning metric based on information about the extent of problems that respondents experience in a range of functioning domains. And it is conditional on the data necessary for constructing a corresponding metric: either a functioning measurement instrument or a selection of item/question set(s) that assess relevant functioning information targeting the intensity of functioning problems by means of ordered response options. Psychometric analysis is then needed to confirm the measurement properties of the functioning metric. Based on a functioning metric, individual’s functioning levels can be expressed in terms of a score (ideally interval-scaled). Further, the functioning of populations can be expressed in terms of a functioning indicator, i.e., a summary statistic of individual functioning scores, such as mean or median [12]. WHO has a long tradition of collecting functioning data to develop metrics of functioning that enable measurement and comparison of the health of populations [13–16]. And since 2014, WHO has introduced a standard valid and reliable survey to collect information on the functioning and disability of populations called “Model Disability Survey” (MDS) [12], whose brief version [17] can be easily added to existing household surveys.

Although information on functioning is already collected in Switzerland through population health surveys, household surveys and cohort studies, the potential to generate a functioning metric and indicator has not been fully exploited yet. We argue in the present study that this is so for two main reasons: first, functioning is not yet fully recognized as the third health indicator that complements mortality and morbidity, and second, there is no overview of either the functioning information collected or the comparability of this data across different sources. This study aims to identify official sources that routinely collect functioning data for the general and the ageing population in Switzerland and to provide an overview of the existing data and its comparability. Additionally, it aims to assess the extent to which data across sources is suitable for developing a functioning metric and indicator that can be used to estimate the need for health services, such as rehabilitation or LTC. This approach makes use of existing data without the need to change current collection tools. The overview is expected to facilitate future efforts to build a functioning metric using Swiss data and to inform the design of future data collection by ensuring that the minimum information needed to construct a functioning metric and indicator is collected in a standardized manner.

## Methods

### Identification of Data Sources

An iterative search was performed to identify official sources used to routinely collect functioning data of the general and the ageing populations in Switzerland. The search was conducted in October and November 2022 through PubMed, and official websites such as the Swiss Federal Statistical Office (Bundesamt für Statistik, FSO) [18], the Federal Office of Public Health (Bundesamt für Gesundheit, FOPH) [19]. For the literature search on PubMed, key terms such as “functioning” OR “disability” AND “cohort study” OR “longitudinal data” OR “health data” AND “elderly” OR “ageing population” AND “Switzerland” OR “swiss” were used. The criteria for including or excluding a data source are described in Table 1.

**TABLE 1:** Inclusion and exclusion criteria for data sources selection and for the extraction of items of interest. (Overview of available functioning data in Switzerland: supporting the use of functioning as a health indicator alongside mortality and morbidity, Switzerland, 2022–2024).

**Inclusion and exclusion criteria for the data sources selection**
*Inclusion criteria* * *- Data sources are official and ongoing/active, and * *- data is routinely collected in Switzerland and available in the public domain, and * *- data sources include cohorts of the ageing population and/or the general population, and * *- data sources focus on health from the perspective of functioning as understood in the ICF (including sources that contain specific modules/panels on functioning or disability among other health topics), and * *- language of the data sources should be English or an official language of Switzerland (German, French, or Italian)
*Exclusion criteria* * *- Data sources are not official, or * *- data is not routinely collected in Switzerland (e.g., surveys designed only for a specific study), or * *- data sources include only specific populations (e.g., diseases-specific data collection exercises), or * *- data sources are looking exclusively at environmental determinants of health
**Inclusion and exclusion criteria for the extraction of items of interest**
*Inclusion criteria* * *- At least one of the main or the additional concept(s) of the item belongs either to a component of functioning and the corresponding item describes/asks about the expression or extent of a problem, or it belongs to environmental factors; and * *- the perspective adopted in the item is descriptive (performance or capacity), need (of assistance) or dependency; and * *- the categorization of response options of the item is intensity, frequency, statement or confirmation/agreement
*Exclusion criteria* * *- The main and the additional concept(s) of the item do not belong to the ICF, or * *- the perspective adopted in the item is appraisal, or * *- the categorization of response options of the item is duration or qualitative attributes

### Mapping Process

Prior to the mapping process, the ICF Research Branch, which maintains a collection of linking tables from various research studies available upon request, was contacted regarding existing tables of the identified data sources. If they had already been linked to the ICF, they were checked for new content or updates (questionnaires, modules, items) since the last linking. Generally, if different versions or data collection periods (so-called “waves”) of a data source were available, the most recent version/wave was used for the mapping and content analysis. The sources or updates were then linked by pairs of professionals trained in the ICF linking rules [20]. The linking process included the following steps.

#### Step 1 Extraction of Items

A list of items (i.e., questions) of interest was developed for each data source. For each item, the item identifier, exact wording and instructions, response options, perspective adopted (descriptive, appraisal, or need or dependency), categorization of response options (intensity, frequency, duration, confirmation or agreement, or qualitative attributes) [20] were independently screened and extracted by two investigators (referred to as “linkers”) according to the specific inclusion and exclusion criteria shown in Table 1. Corresponding inclusion and exclusion criteria were designed to extract potentially useful items to generate a functioning metric and relevant determinants of functioning in the population of the respective survey. The two linkers compared and discussed the items each extracted until they reached consensus on the items to include in the linking process. A third investigator was consulted to resolve continued disagreement on individual items to include in the final item list to link to the ICF.

#### Step 2 ICF Linking

The ICF linking of the extracted items from step 1 was conducted using ICF linking rules [20]. As with the item extraction, two linkers independently extracted the adopted perspective, main and additional concept(s) contained in the items selected in step 1 and linked them to the most accurate ICF categories. Again, a third ICF-trained investigator was consulted to resolve any discrepancies. Note that an item may be coded with several ICF categories, depending on the number of concepts or response options that belong to different components or domains.

### Content Analysis

The final linking tables were compared in terms of the reported ICF categories using the full MDS as a reference. As the MDS was developed based on the ICF and is the current standard recommended by the WHO to measure disability and functioning of populations [17], we considered it suitable to provide a reference list of essential ICF categories for comparison. The full MDS [21] was used for the functioning components (FC), which are the components that are used to develop a potential functioning metric. To compare the environmental factors (EF), the determinants of functioning, the brief MDS [22] was used as a reference list of essential and relevant ICF categories. The linking table of the full MDS was available through the ICF Research Branch. For the brief MDS linking, items were extracted from the full MDS and recoded where necessary (Supplementary Table S1).

## Results

### Identification of Sources

**FIGURE 1:**
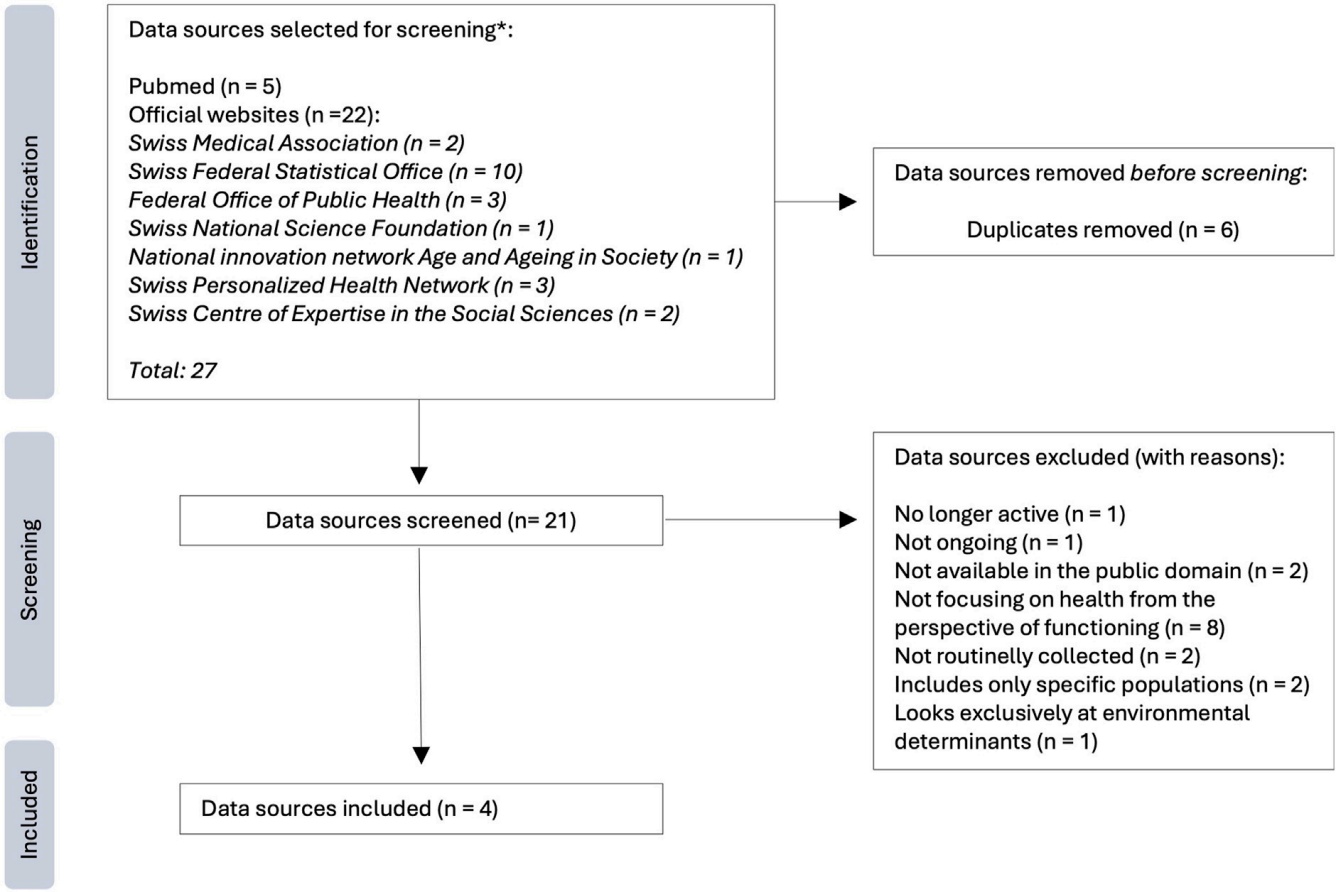
Flowchart of the identification and selection of the data sources. (Overview of available functioning data in Switzerland: supporting the use of functioning as a health indicator alongside mortality and morbidity, Switzerland, 2022–2024) Notes: *The numbers shown correspond to the identified sources that were selected for detailed examination and screening according to our criteria, they do not correspond to the results retrieved from the full search performed; the results of the full search strategy are shown in Supplementary Tables S2, S3 of the Supplementary File 1.

Figure 1 shows the results of the process of the selection of the data sources. Out of 21 screened data sources, four were included: the Swiss part of the Survey of Health, Ageing, and Retirement in Europe (SHARE) [23], the Swiss Health Survey (Schweizerische Gesundheitsbefragung, SHS) [24], the Lausanne cohort 65+ (Lc65+) [25], and the Swiss Household Panel (SHP) [26]. A complete list of all searched institutions/websites and data sources can be found in the Supplementary Tables S2, S3.

Table 2 shows a comparison of the characteristics of these data sources and gives detailed information about which questionnaires were used in this study. The questionnaires of SHARE and SHP were available in English versions. The questionnaires of SHS and Lc65+ were translated from French to English, as they were not available in English.

**TABLE 2:** Overview of the comparison of the characteristics of the included data sources. (Overview of available functioning data in Switzerland: supporting the use of functioning as a health indicator alongside mortality and morbidity, Switzerland, 2022–2024).

**Data source’s name**	Swiss Survey of Health, Ageing and Retirement in Europe	Swiss Health Survey	Lausanne cohort 65+	Swiss Household Panel

**Abbreviation**	SHARE	SHS	Lc65+	SHP

**Development and promotion**	Europe: European Strategy Forum on Research Infrastructures (ESFRI) Country specific: Swiss Centre of expertise in the social sciences of the University of Lausanne (FORS center) and University of Lausanne	Federal Office of Public Health (Bundesamt für Gesundheit, FOPH)	Center for Primary Care and Public Health (Unisanté)	Swiss Centre of Expertise in the Social Sciences of the University of Lausanne (FORS center)

**Purpose**	To study the impact of health, social, economic and environmental policies over the life-course of Swiss citizens [27]	To provide indicators on the state of health, health-related behavior and use of medico-social services, which serve as a basis to plan and evaluate health policy strategies [28]	To gain knowledge of the determinants, the development and the consequences of age-related frailty [25, 29]	To monitor social change, such as the dynamics of changing living conditions in the population of Switzerland [30]

**Scope**	Collects data on the same individuals (with refreshments) on social, economic, and health aspects, ageing, and retirement of individuals in Switzerland as part of a larger European research project [27]	Collects data on various aspects of public health, including lifestyle behaviors, health conditions, and healthcare services utilization [28]	Collects anthropometric measurements and data on the same individuals (with refreshments) on various aspects of the ageing process, including psychosocial characteristics, health, physical and cognitive performance [29]	Collects data on the same households and individuals (with refreshments) on various aspects of household composition, living conditions, health, socioeconomic status, employment, and other socio-demographic factors [30]

**Topics** (main modules)	Social networks, physical health, mental health, behavioral risks, cognitive function, healthcare, walking speed, chair stand, grip strength, social support, housing, activities and expectations [31]	Health status, use of medical devices, personal and social resources, mental health, health behavior, preventive medicine, living conditions [28]	Health, energy and wellbeing, memory, concentration, physical activity, mobility, activities of daily living, environment and living situation, professional and voluntary activities, intergenerational relationships [29]	Household and family, health, social origin, education, employment, income, social participation, networks, leisure, politics, values, religion, and psychological scales [30]

**Target population**	Individuals aged at least 50 years old, along with their partners of any age, who have their regular domicile in Switzerland, excluding hospitalized or incarcerated persons, persons that are out of the country during the entire survey period, or that do not speak the country’s languages [31]	Non-institutionalized individuals aged at least 15 years old, who are permanent residents of Switzerland [28]	Individuals aged at least 65 years old, who are non-institutionalized residents of the city of Lausanne, Switzerland [29]	Members of private households who are non-institutionalized individuals aged at least 14 years old [30]

**Study design**	Multinational longitudinal panel study	Nationwide cross-sectional study	Population-based longitudinal study with cohort-sequential design	Annual longitudinal household panel study

**Sample size and structure**	Size of the baseline/refreshment samples: around 4,000 individual units. (Varies across waves.) [32]	Varies between approximately 13,000 and 22,000 people, depending on the year of the survey [33]	Around 1,500 individual units per sample/year of survey [25]	Comprises four samples: the SHP_I (5,074 households and 7,799 individuals interviewed first in 1999), and the refreshment samples: the SHP_II (2,538 households and 3,654 individuals interviewed first in 2004), the SHP_III (3,989 households and 6,090 individuals interviewed first in 2013) and the SHP_IV (4,380 households and 7,557 individuals interviewed first in 2020) [30]
**Sampling strategy**	Stratified simple random sampling for Switzerland (the sampling strategy is country specific). The selection unit is the household (with at least one person aged 50 or older) [32]	Multistage probability sample drawn of the entire Swiss population after stratification by geographic region [28]	Simple random sampling method using the Residents’ Register of the canton Vaud, Switzerland [29]	Stratified random sampling method. The four random samples are stratified by the seven major statistical regions of Switzerland [30]

**Survey language**	German, French, Italian	German, French, Italian	French	German, French, Italian, and English

**Survey cycle**	Since 2004, every 2 years (except in the year 2008 – wave 3)	Since 1992, every 5 years	Since 2004, every year (written questionnaire) and every 3 years (interview-examination)	Since 1999, every year

**Collection mode**	In-person interviews utilizing computer-assisted personal interviewing	Computer-assisted telephone interview or face-to-face conversation and a written follow-up questionnaire	Postal written questionnaire and interview-examination	Telephone interview or face-to-face interview. Since 2018, there is a web-based version

**Assessment**	Self-reported or proxy	Self-reported	Self-reported and examination	Self-reported or proxy

**Types of questionnaires**	SHARE core interview SHARELIFE Corona Surveys 1 & 2	Interview and written questionnaire	Postal written questionnaire and interview-examination	Household grid questionnaire to assess household composition, a household questionnaire, an individual questionnaire and a proxy questionnaire

**Waves or versions used for mapping**	Wave 8 (year 2019/2020)	SHS 2017	Version of year 2023	Core interview of wave 22 (year 2020/2021) and rotating modules of waves 18, 21, 22, and 23

**Questionnaires used for mapping**	SHARE core interview	Interview and written questionnaire	Postal written questionnaire and interview-examination	Individual, household, and proxy questionnaire

**Reasons for using specific waves and questionnaires**	Most recent wave available at the time of data analysis. Special dedicated questionnaires with special modules, such as the SHARELIFE questionnaire, conducted for waves 3 and 7, and two additional single wave studies, the SHARE Corona Surveys 1 & 2, added in 2020 and 2021, were not considered for the purpose of this study	The final version of the SHS 2022 was not available at the time of data analysis. SHS 2017 was the most recent one	Most recent questionnaires and interview-examination records at the time of data analysis	Most recent wave available at the time of data analysis. The rotating modules were included to ensure that all items of the most recent waves were included. The household grid questionnaire was not considered for the purpose of this study, as it assesses household composition

**Link to the source’s documentation**	https://www.share-datadocutool.org/study-units/view/1	https://www.bfs.admin.ch/bfs/fr/home/statistiques/sante/enquetes/sgb.html	https://www.lc65plus.ch/en/content/research-documents	https://forscenter.ch/projects/swiss-household-panel/documentation/

### Mapping Process

Because of the length of the included sources and considering the lessons learned after screening the first tool, the planned linking process was adjusted. One reviewer (BM) would pre-select modules and items independently before step 1, after which the extraction and linking would be performed. The extraction (step 1) and linking (step 2) of items were performed by MS and JK for SHARE, and by MS and BM for the remaining tools. Discrepancies in both steps were discussed with a third linker (BM for SHARE and JH for the other tools).

Moreover, while extracting the items of interest (i.e., items to include for the linking process), the investigators found that the inclusion criterion that items of FC should describe the expression or extent of a problem was open to interpretation. To ensure data quality, the linking results underwent a final review and consistency check by BM, JH, and CS. The final linking tables can be found in the Supplementary Table S4 of the Supplementary File 2.

Previous data collection waves of the SHS and the SHP had been linked to the ICF and were available through the ICF Research Branch. Before step 1, previously linked items from SHS 2012 and SHP wave 18 were merged with new items from SHS 2017 and SHP wave 22, respectively. The merging included eliminating items present in previous sources but no longer in the most recent versions. For previously linked items, the reviewers checked the concepts, perspective adopted, categorization of response options and the ICF categories and revised them when necessary. Revisions included adding missing information or specification of ICF categories to the most accurate category.

In total, the four sources contained 71 items (out of 863 items identified in total) about FC, of which around 70% were coded as *body functions*, and 30% as *activities and participation*; and 61 items about EF. Most items linked across sources have a descriptive perspective and confirmation or agreement as response option. Items had various timeframes, ranging from “in the last 12 months” (Lc65+) to “at the time of the interview” (all sources).

### Content Analysis: Results for the Functioning Components

Table 3 shows an overview of the ICF categories covered in the identified sources, using the MDS as a reference. SHARE and Lc65+ are the sources with the most categories in common with the MDS. The SHP is the source with the least categories in common with the MDS, and the other sources; the SHP only covers five *body functions* and two *activities and participation* categories, and these items, *d850 Remunerative employment* and *d920 Recreation and leisure*, are not present in the other sources.

**TABLE 3:** Comparison of International Classification of Functioning, Disability and Health categories covered in the identified data sources that reflect the expression or extent of a functioning problem, using the World Health Organization’s Model Disability Survey as a reference. Categories that belong to the brief Model Disability Survey are in bold. Categories covered in all sources are highlighted in dark grey. Categories covered by 3 sources are highlighted in light grey. (Overview of available functioning data in Switzerland: supporting the use of functioning as a health indicator alongside mortality and morbidity, Switzerland, 2022–2024).

ICF category in MDS		Data source			
		SHARE	SHS	Lc65+	SHP
**Body functions**					
**b134**	**Sleep functions**	**x**	**x**	**x**	**x**
**b1300**	**Energy level**	**x**	**x**	**x**	**x**
**b140**	**Attention functions**	**x**	**x** ^a^ ***(b1400 Sustaining attention)***	**x** ^a^ **(*b1401 Shifting attention*)**	
**b144**	**Memory functions**	**x**		**x** ^a^ ***(b1441 Long-term memory)***	
**b152**	**Emotional functions**	**x**	**x**	**x**	**x**
b1646	Problem Solving				
**b210**	**Seeing functions**	**x** ^a^	**x**		
b21000	Binocular acuity of distant vision	x			
b21002	Binocular acuity of near vision	x			
b230	Hearing functions	x			
**b280**	**Sensation of pain**	**x**	**x** ^a^	**x** ^a^	**x**
b440	Respiration functions			x	
b450	Additional respiratory functions			x^a^ *(b4501 Functions of coughing)*	
b455	Exercise tolerance functions		x	x	x
**Activities and participation**					
d159	Basic learning, other specified and unspecified (learning a new task)				
**d240**	**Handling stress and other psychological demands**				
d2401	Handling stress				
d3	Communication	x^a^ *(d3600 Using telecommunication devices; d325 Communicating with - receiving - written messages)*	x^a^ *(d360 Using communication devices and techniques; d330 Speaking)*	x^a^ *(d360 Using communication devices and techniques)*	
d329	Communicating - receiving, other specified and unspecified (being understood)				
d349	Communicating - producing, other specified and unspecified (being understood)				
d350	Conversation		x		
d4104	Standing	x		x	
d4154	Maintaining a standing position				
d4300	Lifting				
d440	Fine hand use	x		x^a^ *(d4400 Picking up)*	
**d450**	**Walking**	**x^a^**	**x^a^**	**x^a^**	
d4500	Walking short distances	x	x	x	
**d4501**	**Walking long distances**		**x**		
d455	Moving around				
**d4551**	**Climbing**				
**d470**	**Using transportation**	**x**	**x**		
d498	Mobility, other specified (getting where you want to go)				
**d5**	**Self-care**	**x^a^**	**x^a^**	**x^a^**	
d5204	Caring for toenails				
**d530**	**Toileting**	**x**	**x**	**x**	
d550	Eating	x	x	x	
**d570**	**Looking after one’s health**	**x^a^ (*d5702 Maintaining one’s health)***		**x^a^ (*d5702 Maintaining one’s health)***	
**d640**	**Doing housework**	**x^a^ *(d6400 Washing and drying clothes and garments)***	**x^a^ *(d6400 Washing and drying clothes and garments)***	**x**	
d649	Household tasks, other specified and unspecified				
d660	Assisting others				
d730	Relating with strangers				
d7500	Informal relationships with friends				
d770	Intimate relationships				
d779	Particular interpersonal relationships, other specified and unspecified (getting along with people who are close including family and friends)				
**d820**	**School education**				
d839	Education, other specified and unspecified (getting a formal or informal education)				
d8450	Seeking employment				
**d850**	**Remunerative employment**				**x**
d860	Basic economic transactions	x	x	x	
d898	Major life areas, other specified (day-to-day work or school)				
**d910**	**Community life**				
d920	Recreation and leisure				x
**d930**	**Religion and spirituality**				
d950	Political life and citizenship				

Notes: More than one item can be included in each category. One item can be coded with several ICF, categories, depending on the number of concepts and response options.

^a^ Includes items with an ICF, code of a more detailed level. In the comparison, higher level categories include all items coded to more detailed levels. When the more detailed code is not on the table, the ICF, category is added to the row of the respective data source in parenthesis.

ICF: International Classification of Functioning, Disability and Health; MDS: Model Disability Survey; SHARE: Survey of Health, Ageing and Retirement in Europe; SHS: Swiss Health Survey; Lc65+: Lausanne cohort 65+; SHP: Swiss Household Panel.

Four ICF categories are covered across all sources (Table 3, highlighted in dark grey) and they all belong to *body functions*: *b134 Sleep functions*, *b1300 Energy level*, *b152 Emotional functions*, and *b280 Sensation of pain*. These categories belong to both the full MDS and the brief MDS (Table 3, bold). Regarding *activities and participation*, no ICF categories are covered across all sources. However, *b140 Attention functions*, *d3 Communication*, *d450 Walking* and *d4500 Walking short distances*, *d5 Self-care*, *d530 Toileting*, *d550 Eating*, *d640 Doing housework,* and *d860 Basic economic transactions* are included in all the data sources, except for the SHP (Table 3, highlighted in light grey) while *b455 Exercise tolerance functions* is included in SHS, Lc65+, and SHP. Of those, walking, self-care, toileting, doing housework belong also to the brief MDS. Categories of the full and brief MDS that none of the sources cover include, for example, handling stress and other psychological demands.

Table 4 compares the wording of the items and their response options for the categories common to the MDS in all sources. Overall, Lc65+ has more items covering the common domains than the other sources, followed by SHS. Most items use frequency as response option, while Lc65+ and SHS focus more on response options that assess intensity.

**TABLE 4:** Comparison of the wording of the items and their response options for the International Classification of Functioning, Disability and Health categories of the functioning components common to the Survey of Health, Ageing and Retirement in Europe; Swiss Health Survey; Lausanne cohort 65+, and Swiss Household Panel, using the Model Disability Survey as a reference. (Overview of available functioning data in Switzerland: supporting the use of functioning as a health indicator alongside mortality and morbidity, Switzerland, 2022–2024).

ICF code				
Data source	Item identifier and wording	Response option	Categorization of response option
**b134 Sleep functions**			
SHARE	*MH007_Sleep* Have you had trouble sleeping recently?	Trouble with sleep or recent change in pattern; No trouble sleeping	Confirmation

SHS	^a^ *Q21.00* Tell me if you have had [the following complaint] over the past 4 weeks, a little or a lot. (If currently taking a medicine for a complaint, it means you have that complaint) 5) Difficulty falling asleep or staying asleep	Not at all; A little; Strongly	Intensity

	^b^ *Q51* During the last 2 weeks, how often have you felt affected by the following complaints: Please tick the appropriate answer for each line! c) Difficulty falling asleep or staying asleep, or increased sleep?	Never; Several days; More than half the days; Almost every day	Frequency

	^b^ *S0500* How often do you … a) Have difficulty falling asleep? b) Sleep restlessly? d) Wake up too early in the morning? c) Wake up several times during the night?	Always, Sometimes; Rarely; Never	Frequency

Lc65+	*16.* During the last 4 weeks, have you had difficulty falling asleep or insomnia?	Not at all; A little; A lot	Intensity

SHP	^b^ *118.* During the last 4 weeks, have you suffered from any of the following disorders or health problems? Difficulty sleeping, or insomnia	Not at all; Somewhat; Very much	Confirmation
**b1300 Energy level**				
SHARE	*MH013_Fatigue* In the last month, have you had too little energy to do the things you wanted to do?	Yes; No	Confirmation

	*AC023_FullEnerg* How often do you feel full of energy these days?	Often; Sometimes; Rarely; Never	Frequency

SHS	^b^ *Q21.00* Tell me if you have had [the following complaint] over the past 4 weeks, a little or a lot. (If currently taking a medicine for a complaint, it means you have that complaint) 2) General weakness, fatigue, lack of energy	Not at all; A little; Strongly	Intensity

	^b^ *Q51* During the last 2 weeks, how often have you felt affected by the following complaints: Please tick the appropriate answer for each line! (Please select the appropriate answer for each line!) d) Fatigue or feeling of having no energy?	Never; Several days; More than half the days; Almost every day	Frequency

	^b^ *S6400* How have you felt in the last 4 weeks? a) Full of life b) Full of energy c) Exhausted d) Tired	Always; Mostly; Sometimes; Rarely; Never	Frequency

Lc65+	*14.* During the past 4 weeks, have you had a generalized feeling of weakness, weariness, or lack of energy?	Not at all; A little; A lot	Intensity

	*22.* In the past 4 weeks, how often have you felt strong, energetic and optimistic?	Always; Very often; Often; Sometimes; Rarely; Never	Frequency

	*6E b.* In the last 4 weeks, were there any moments when you felt energized?	Permanently, Very often, Sometimes, Rarely, Never	Frequency

SHP	*139.* Are you often plenty of strength, energy and optimism, if 0 means “never” and 10“always?”	0“never” - 10“always"	Frequency

	*117.* During the last 4 weeks, have you suffered from any of the following disorders or health problems? General weakness, weariness, or lack of energy	Not at all; Somewhat; Very much	Intensity
**b152 Emotional functions**			
SHARE	*MH002_Depression* In the last month, have you been sad or depressed? If participant asks for clarification, say “by sad or depressed, we mean miserable, in low spirits, or blue”	Yes; No.	Confirmation

SHS	^b^ *2,500.* I am now going to read you different feeling states. Please tell me each time whether you have felt this way always, most of the time, sometimes, rarely, or never in the last 4 weeks 1) Very nervous 2) So depressed or upset that nothing could cheer you up 4) Discouraged and depressed 5) Happy	Always; Mostly; Sometimes; Rarely; Never	Frequency

	^b^ *Q51.* During the last 2 weeks, how often have you felt affected by the following complaints: Please tick the appropriate answer for each line! a) Little interest or pleasure in your activities? b) Feeling down, melancholy, or hopeless?	Never, Several days, More than half the days, Almost every day	Frequency

Lc65+	*17.* During the last 4 weeks, have you often felt sad, depressed or discouraged?	Yes; No	Confirmation

	*18*. During the past 4 weeks, have you often felt a lack of interest or pleasure in your usual activities?	Yes; No	Confirmation

	*19*. During the last 4 weeks, have you often felt worried and anxious?	Yes; No	Confirmation

	*4E b.* In the past 4 weeks, because of your emotional state (such as feeling sad, nervous or depressed), have you done what you needed to do with less care and attention than usual?	Permanently; Very often; Sometimes; Rarely; Never	Frequency

	*6E c.* In the past 4 weeks, have there been times when you have felt sad and depressed?	Permanently; Very often; Sometimes; Rarely; Never	Frequency

SHP	*138*. Do you often have negative feelings such as having the blues, being desperate, suffering from anxiety or depression, if 0 means “never” and 10 “always"?	0“never” - 10 “always"	Frequency

	*140*. How often have you felt stressed during the last month?	Never; Almost never; Sometimes; Fairly often; Very often	Frequency
**b280 Sensation of pain**				
SHARE	*PH084_TroubledPain*. Are you troubled with pain?	Yes; No.	Confirmation

	*PH085_PainLevel.* How bad is the pain most of the time?	Mild; Moderate; Severe	Intensity

SHS	^b^ *Q21.00* Tell me if you have had [the following complaint] over the past 4 weeks, a little or a lot. (If currently taking a medicine for a complaint, it means you have that complaint) 1) Back or low back pain 3) Pain or feeling of pressure in the abdomen 6) Headache, pressure in the head or facial pain 8) Pain or pressure in the chest area 10) Pain in the shoulders, neck and/or arms	Not at all; A little; Strongly	Intensity

Lc65+	^b^ *5* Have you been bothered, for at least 6 months, by … when you exert yourself	Joint pain in the legs, shoulders, arms or hands; back pain; chest pain	Confirmation

	*18* In the past 4 weeks, to what extent has physical pain limited you in your work or limited you in your work or domestic activities?	Not at all; A little bit; Moderately; Very much; A lot	Intensity

	*5E a.* In the past 4 weeks, how much have you been limited in your work or home activities by physical pain?	Not at all; A little bit; Medium, Many; Very much	Intensity

	*71E.* Do you have pain or sensitivity in your gums or teeth when you chew food?	No, none; Yes, but light; Yes, a lot	Intensity
SHP	^b^ *116* During the last 4 weeks, have you suffered from any of the following disorders or health problems? Bad back or lower back problems	Not at all; Somewhat; Very much	Intensity

	^b^ *119* During the last 4 weeks, have you suffered from any of the following disorders or health problems? Headaches or facial pain	Not at all; Somewhat; Very much	Intensity

Notes: More than one item can be included in each category, and one item can be coded with several ICF, categories, depending on the number of concepts and response options. Higher level categories include all items coded to more detailed levels.

^a^ Items with an ICF code of a more detailed level included in a less detailed category.

^b^ Only relevant response options are shown.

Note that for the SHP, the items about sensation of pain and sleep functions do not have a different formulation of the question; instead, they are grouped under the umbrella of health problems and disorders, and the ICF, category here refers to the concept in the response option itself. The same happens for specific items in SHS (Q21.00 and Q51).

ICF: International Classification of Functioning, Disability and Health; MDS: Model Disability Survey; SHARE: Survey of Health, Ageing and Retirement in Europe; SHS: Swiss Health Survey; Lc65+: Lausanne cohort 65+; SHP: Swiss Household Panel.

Table 4 shows that the wording of the items is different across sources and that the types of response options are mixed. Commonly assessed topics include insomnia and difficulty falling asleep for the category *b134 Sleep functions*; lack or plenty of energy for the category *b1300 Energy level*; feelings about sadness, depression or anxiety for the category *b152 Emotional functions*; and back pain for the category *b280 Sensation of pain*. Except for SHP which used a continuous scale (0“Never” – 10 “Always”), all other sources used a 4-point response scale (with variations of “Always, Often, Sometimes, Rarely, Never”). The response options for assessing intensity of problems vary greatly, also within the same tool (in Lc65+: “Not at all; A little bit; Medium; Many; Very much” and “No, none; Yes, but light; Yes, a lot”), depending on the formulation of the question. The category with the highest number of items with intensity of problems as response option is *b280 Sensation of pain*, while for *b152 Emotional functions* there is no item with intensity as an option. *b152 Emotional functions* and *b1300 Energy level* are assessed more often with response options that reflect frequency.

A similar comparison for the common categories in three tools can be found in the Supplementary Table S5.

### Content Analysis: Results for the Determinants of Functioning

All the sources include questions on the EF. Most items reflect a descriptive perspective and confirmative response option (Supplementary Table S6). Exceptions are items coded as *e3 Support and relationships*, which often reflect a need or dependency perspective. Table 5 provides an overview of the commonly covered ICF categories in each tool, with the brief MDS as a reference. SHARE and SHS are the tools with the most categories in common with the brief MDS. The Lc65+ is the tool with fewest categories in common with the brief MDS, and with the other tools.

**TABLE 5:** Comparison of International Classification of Functioning, Disability and Health categories of environmental factors covered in the Brief Model Disability Survey and in the data sources. Categories covered by 3 sources are highlighted in light grey. (Overview of available functioning data in Switzerland: supporting the use of functioning as a health indicator alongside mortality and morbidity, Switzerland, 2022–2024).

		Data source			
ICF category in MDS		SHARE	SHS	Lc65+	SHP
**Environmental factors**				
e1	Products and technology	X^a^		X^a^
e115	Products and technology for personal use in daily living	X			
e120	Products and technology for personal indoor and outdoor mobility and transportation	X		X
e125	Products and technology for communication			X
e155	Design, construction and building products and technology of buildings for private use	X			
e3	Support and relationship	X^a^	X^a^		X^a^
e310	Immediate family	X	X		X
e320	Friends	X	X		X
e325	Acquitances, peers, colleagues, neighbours and community members	X	X		X
e4	Attitudes		X^a^		

Notes: More than one item can be included in each category, and that one item can be coded with several ICF, categories, depending on the number of concepts and response options.

^a^ Includes items with an ICF, code of a more detailed level.

ICF: International Classification of Functioning, Disability and Health; MDS: Model Disability Survey; SHARE: Survey of Health, Ageing and Retirement in Europe; SHS: Swiss Health Survey; Lc65+: Lausanne cohort 65+; SHP: Swiss Household Panel.

Most tools (SHARE, SHS, and SHP) overlap in items belonging to the chapter *e3 Support and relationships*. The chapter *e4 Attitudes* is only covered by the SHS, while Lc65+ focuses only on products and technology, but not on support and relationships.

## Discussion

This study identified and examined the content and comparability of existing official sources that collect functioning data in Switzerland, using the current WHO functioning and disability survey as a reference framework. In total, we identified four sources that routinely collect data on functioning: SHARE, SHS, Lc65+, and SHP. They all share several conceptually equivalent items about specific functioning categories, namely four *body functions* – sleep functions, energy and drive, emotional functions, and sensation of pain – while three of them (SHARE, SHS, and Lc65+) have nine categories in common: attention functions, communication, walking, walking short distances, self-care, toileting, eating, doing housework and economic transactions. Across sources, the response options for assessing frequency of problems are similar while response options assessing intensity of problems vary greatly, also within the same tool. Regarding the determinants of functioning, all the sources collect information about EF, namely about support and relationships.

To our knowledge, this is the first study to compare functioning data across official population-based data sources in Switzerland. Different studies about content mappings and analyses using the ICF as a framework exist in different contexts. However, comparisons between them are generally difficult due to the specific goals shaping each analysis’s execution and interpretation. Instead of focusing on the mapping and comparison of specific instruments and/or conditions or sub-populations in clinical settings [34–36], our study focused on population data sources. We applied the ICF linking rules to compare population-based data from different sources similarly to previous work [37], but our comparison focused on the availability of functioning information suitable for the construction of a functioning metric and indicator.

Our findings show that Switzerland has readily available routinely collected functioning data that can serve as the basis for creating metrics of functioning. To do that, it is important to have a suitable collection of items that reflect relevant categories of functioning and ask about the extent or presence of problems, as functioning is understood as a matter of degree ranging from no to extreme problems. Different approaches to developing such metrics are possible. One approach involves using items from one setting (data source) and creating a corresponding metric – a particular functioning metric. This metric is independent of the development data and can be applied in similar settings. Another approach involves selecting items that are common across tools and constructing a common metric – a common functioning metric. Our study demonstrates that functioning data is available in Switzerland for creating particular functioning metrics and indicators but that a higher level of overlap across sources in terms of ICF categories and response options focusing on intensity of problems, and not on frequency, would be needed to create a common functioning metric.

Evidence on the feasibility of the construction of functioning metrics and corresponding overall scores is broad. In the general population, a common functioning metric has been developed and used to compare the health of the English and the Americans by challenging the standard practice of counting the prevalence of chronic diseases to determine how healthy a population is [38]. Importantly for policymakers, the English have better health than Americans if prevalence of disease is used but the advantage disappears almost completely when a functioning metric is used [38]. Functioning has also been used to study global patterns of ageing [39] and to build and model healthy ageing trajectories and gather a better understanding of their determinants [40]. In these studies, psychometric analyses according to Item Response Theory and Rasch Measurement Models were performed to estimate the functioning metrics and scores. Moreover, it has been demonstrated that a valid and reliable functioning metric and overall scores can be built even using a minimal selection of functioning domains [11, 41]. The so-called minimal ICF set requires information about the level of problems in energy and drive functions, emotional functions, pain, carrying out daily routine, walking and moving around [11]; variables that are available in three of the four sources examined in our study. Some of these categories pertain to ADL, which are traditionally used to measure functional status in older persons, but importantly, also to body functions such as pain, fatigue, and emotional functions.

Although common ICF categories have been identified across sources, there are several other considerations for a successful development of a common functioning metric. From a psychometric analysis perspective, successful equating across sources depends, for example, on the proportion of common items, the location of the items on the continuum, the quality of the data (missing values, homogeneity), the fit of the items, and the targeting of the scales [42]. From a data characteristics perspective, as functioning is understood as a matter of degree (ranging from low to high levels) data about the presence or intensity of problems is essential. For example, the frequency with which an activity is performed does not necessarily indicate that a person does or does not have a problem in functioning. Therefore, building functioning metrics is conditional on having items that can identify people with mild to severe problems. The variability in the wording of items and in response options for the selected items in our study, as well as items targeting frequency rather than intensity of problems, both within and across tools, is challenging when building a common metric.

If the use of a functioning indicator for Switzerland is planned, two minor changes to the structure of the current data collection (similar to how socio-demographic variables are standardized [43]) might be considered to achieve a comparable minimum set of functioning items across surveys. First, converting frequency response options into intensity and adhering to consistent descriptors for intensity levels, such as “Not at all,” “A little bit,” “Medium,” “Many,” and “Very much,” is an essential step for measuring functioning on an intensity-of-problems continuum. Secondly, it is necessary to transform the response options from confirmation (the problem is present or not) to intensity, ideally using more than two options, to allow for the expression of mild and moderate functioning problems [44]. These minor changes would facilitate meaningful cross-tools comparisons and the development of functioning metrics. Lastly, given that Switzerland has plans to establish a large population-based cohort [45], it would be important to ensure that at least the domains of the minimal ICF generic set [11] are covered to be able to generate a common functioning metric that makes it possible to compare general and specific populations.

### Limitations

This study has several limitations. First, although we performed an iterative search, we may have missed Swiss data sources that fit our criteria. Moreover, potentially important sources might have been excluded due to the lack of access to the data at the time of the search. Secondly, the identified sources are developed and conducted by different actors in Switzerland, and have distinct purposes, reflected in their scope, topics and target population, which limits direct comparisons. Thirdly, we only included and mapped the most recent waves of the tools’ questionnaires, so we may have missed common items across tools from previous waves. Finally, although previous linking tables from the ICF Research Branch have been checked by the linkers, relying on them may have introduced inconsistencies in the mapping process.

### Conclusion

In conclusion, using the ICF linking rules and the MDS as reference framework, this study provides an overview of the currently available population-based functioning data in Switzerland across different data sources. Our results can inform the development of functioning metrics as well as of a potential common Swiss functioning metric and indicator based on official data collected directly and routinely from individuals of the general and ageing populations. This benefits researchers and policymakers as a reliable and informative indicator would become available to complement mortality and morbidity data, to support both the estimation of rehabilitation and LTC needs, and to inform the development and monitoring of policies and programs without the need to implement new data collection tools.

## Author’s Note

The paper is part of the cumulative PhD thesis of BM.
