# Supplementary material for: Overview of Available Functioning Data in Switzerland: Supporting the Use of Functioning as a Health Indicator Alongside Mortality and Morbidity
Source: Int J Public Health. 2024 Aug 14;69:1607366. doi: 10.3389/ijph.2024.1607366 (PMC11349544; doi:10.3389/ijph.2024.1607366)
Supplement: Supplementary file 1 [file DataSheet1.zip › Supplementary File 1.docx]

**International Journal of Public Health**

**Supplementary file 1**

**Overview of available functioning data in Switzerland: supporting the use of functioning as a health indicator alongside mortality and morbidity.**

**Table S1.** Brief Model Disability Survey linking table for environmental factors. Adapted from the full Model Disability Survey linking table provided by the International Classification of Functioning, Disability and Health Research Branch. (Overview of available functioning data in Switzerland: supporting the use of functioning as a health indicator alongside mortality and morbidity, Switzerland, 2022-2024)

| **Model disability survey (MDS) brief version questions** | **Full MDS question number** | **Final linking** |
| --- | --- | --- |
| Module 3000A: ENVIRONMENTAL FACTORS |  |  |
| I am going to ask you some general questions about your environment and your social relationships.  I want you to answer the following questions on a scale from 1 to 5, where 1 means very easy and 5 means very hard. INTERVIEWER: USE show card B001. |  |  |
| B3001 Do places where you socialize and engage in community activities make it easy or hard for you to do this? 1 2 3 4 5 8 98 | I3003 | nd-e Environmental factors, not defined (community-related) |
| B3002 Do the shops, banks and post office in your neighborhood make it easy or hard for you to use them? 1 2 3 4 5 8 98 | I3004 | nd-e Environmental factors, not defined (neighbourhood) |
| B3003 Does the transportation you need or want to use make it easy or hard for you to use it? 1 2 3 4 5 8 98 | I3006 | nd-e Environmental factors, not defined (transport-related) |
| B3004 Does your dwelling, including the toilet, make it easy or hard for you to live there? 1 2 3 4 5 8 98 | I3007 | e155 Design, construction and building products and technology of buildings for private use |
| B3005 Should you need help, how easy is it for you to get help from a close family member (including your partner)? 1 2 3 4 5 98 | I3043 | e310 Immediate family |
| B3006 Should you need help, how easy is it for you to get help from friends and co-workers? 1 2 3 4 5 98 | I3044 | e320 Friends  e325 Acquitances, peers, colleagues, neighbours and community members |
| B3007 Should you need help, how easy is it for you to get help from neighbors? | I3045 | e325 Acquitances, peers, colleagues, neighbours and community members |
| I want you to answer the following two questions on a scale from 1 to 5 where 1 is not at all and 5 means completely. |  |  |
| B3008 Do you make your own choices about your day-to-day life? For example, where to go, what to do, what to eat. 1 2 3 4 5 98 | I3056 | e4 Attitudes |
| B3009 Do you feel that other people respect you? For example, do you feel that others value you as a person and listen to what you have to say? 1 2 3 4 5 98 | I3059 | e4 Attitudes |
|  |  |  |
| Module 3000B: PERSONAL ASSISTANCE AND ASSISTIVE PRODUCTS |  |  |
| B3010 Do you have someone to assist you with your day to day activities at home or outside? 1 YES 5 NO If 5, go to B3012 | I3010 | e3 Support and relationship |
| B3011 Do you think you need additional assistance with your day to day activities at home or outside? 1 YES 5 NO Go to B3013 | I3013 | e3 Support and relationship |
| B3012 Do you think you need someone to assist you? 1 YES 5 NO | I3014 | e3 Support and relationship |
| B3013 Do you currently use any of these assistive products?  INTERVIEWER: USE show card B004 and circle all mentioned products. If 1, go to B3015  1) None  2) Canes or Sticks  3) Crutches, axillary or elbow  4) Orthoses, lower limb, upper limb or spinal  5) Pressure relief cushions  6) Prostheses, lower limb  7) Rollators  8) Standing frames, adjustable  9) Therapeutic footwear; diabetic, neuropathic, orthopedic  10) Tricycles  11) Walking frames or walkers  12) Wheelchair  13) Spectacles; low vision, short distance, long distance, filters and protection  14) White cane  15) Hearing aids  16) Others |  | e1 Products and technology  e125 Products and technology for communication  e120 Products and technology for personal indoor and outdoor mobility and transportation  e115 Products and technology for personal use in daily living |
| B3015 You told me you do not use assistive products. Do you think you need any of these?  INTERVIEWER: USE show card B004 and circle all mentioned products.  1) None  2) Canes or Sticks  3) Crutches, axillary or elbow  4) Orthoses, lower limb, upper limb or spinal  5) Pressure relief cushions  6) Prostheses, lower limb  7) Rollators  8) Standing frames, adjustable  9) Therapeutic footwear; diabetic, neuropathic, orthopedic  10) Tricycles  11) Walking frames or walkers  12) Wheelchair  13) Spectacles; low vision, short distance, long distance, filters and protection  14) White cane  15) Hearing aids  16) Others |  | e1 Products and technology  e125 Products and technology for communication  e120 Products and technology for personal indoor and outdoor mobility and transportation  e115 Products and technology for personal use in daily living |

**Table S2**. List of all searched institutions/official websites, and respective results. (Overview of available functioning data in Switzerland: supporting the use of functioning as a health indicator alongside mortality and morbidity, Switzerland, 2022-2024)

| **Institution/Website** | **Databases** | | **Status (included/excluded)** | **Reason for exclusion** |
| --- | --- | --- | --- | --- |
| Swiss Medical Association (Forum medizinischer Register Schweiz, FMH)  <https://www.fmh.ch> | Swiss National Cohort (SNC) | | Included |  |
|  | Patient Centered Outcome Register (PCOR) | | Excluded | No longer active |
| Swiss Federal Statistical Office (Bundesamt für Statistik, FSO)  <https://www.bfs.admin.ch> | Swiss National Cohort (SNC) | Swiss Federal Census 1990 & 2000 | Excluded | No health or functioning data. |
|  |  | Vital statistics & Mortality data from BEVNAT | Excluded | No health or functioning data. |
|  |  | Structural survey | Excluded | No health or functioning data. |
|  |  | Swiss health survey (SHS) | Included |  |
|  |  | Buildings and Dwellings statistic (BDS) | Excluded | No health or functioning data. |
|  |  | Swiss neighbourhood index (Swiss-SEP) | Excluded | No health or functioning data. |
|  |  | STATPOP | Excluded | No health or functioning data. |
|  | Swiss Health Survey (SHS) |  | Included |  |
|  | State of health of elderly people in institutions (Gesundheitszustand von betagten Personen in Institutionen, EGBI) |  | Excluded | Not routinely collected |
|  | Swiss Survey of Health, Ageing, and Retirement in Europe (SHARE) |  | Included |  |
| Federal Office of Public Health (Bundesamt für Gesundheit, FOPH)  <https://www.bag.admin.ch> | Swiss Health Study (Schweizer Gesundheitsstudie) |  | Excluded | Assesses the impact of chemical substances and noise on health, not focusing on functioning (focus on environmental determinants of health). The study is to be carried out at the national level only from 2023 – not ongoing (at the time of the search). |
|  | Swiss Health Survey (SHS) |  | Included |  |
|  | Swiss Survey of Health, Ageing, and Retirement in Europe (SHARE) |  | Included |  |
| Swiss National Science Foundation (Schweizerischer Nationalfonds, SNF)  <https://www.snf.ch/en/YA2SxeDV03G25gyJ/funding/programmes/longitudinal-studies> | SAPALDIA Cohort on Healthy Aging |  | Excluded | The study focuses on air pollution, lung disease, cardiovascular, and metabolic health.  Not available in the public domain. |
| National innovation network Age and Ageing in Society (AGE-NT)  <https://www.age-netzwerk.ch/en/netzwerk/das-projekt/> | Swiss Survey65+ |  | Excluded | Not routinely collected |
| Swiss Personalized Health Network (SPHN)  <https://sphn.ch> | Lausanne cohort 65+ (Lc65+) |  | Included |  |
|  | Swiss Frailty Network Repository (SFNR) |  | Excluded | A database of routinely collected electronic patient data of patients admitted to acute geriatric care. Not public domain, uses electronic health records (EHRs). |
|  | The Swiss Ageing Citizen Reference (SACR) |  | Excluded | A database/reference cohort of data and biospecimens of existing cohorts focusing on biomarkers of ageing (including blood DNA methylation and brain images). Not focusing on health from the perspective of functioning. |
| Swiss Centre of Expertise in the Social Sciences (FORS)  <https://forscenter.ch> | Swiss Household Panel (SHP) |  | Included |  |
|  | Survey of Health, Ageing, and Retirement in Europe (SHARE) |  | Included |  |

**Table S3**. PubMed search strategy, and respective results. (Overview of available functioning data in Switzerland: supporting the use of functioning as a health indicator alongside mortality and morbidity, Switzerland, 2022-2024)

| **Search** | **Search terms** | **Results (number)** | **Findings**  **(databases)** | **Status (included/excluded)** | **Reason for exclusion** |
| --- | --- | --- | --- | --- | --- |
| #1 | "functioning" AND data AND ageing population AND (swiss OR switzerland) | 46 | Swiss Spinal Cord Injury (SwiSCI) Cohort Study | Excluded | Includes only a specific population |
| #2 | functioning AND (score OR indicator) AND "disability" AND (swiss OR switzerland) AND policy AND (elderly OR ageing) | 23 | The Swiss Multiple Sclerosis Cohort-Study (SMSC) | Excluded | Includes only a specific population |
| #3 | “functioning score” AND (ageing OR elderly) AND switzerland | 8 | - |  |  |
| #4 | ("functioning indicator" OR "functioning score") AND (ageing OR elderly) | 326 | - |  |  |
| #5 | (health-survey OR cohort study) AND (swiss OR switzerland) AND (elderly OR ageing) | 33,727 | Swiss Longitudinal Cohort Study (SWICOS) | Excluded | Focus on diseases/ health conditions (mainly cardiovascular). Not focusing on health from the perspective of functioning. |
|  |  |  | Survey of Health, Ageing, and Retirement in Europe (SHARE) | Included |  |
|  |  |  | Swiss Health Survey (SHS) | Included |  |
| #6 | "secondary use of health data" AND functioning | 5 | - |  |  |
| #7 | "health data" AND switzerland AND (functioning OR disability) | 115 | - |  |  |
| #8 | “cohort study” AND (ageing OR elderly) AND switzerland AND functioning | 1544 | - |  |  |
| #9 | “longitudinal data” AND (ageing OR elderly) AND switzerland AND functioning | 87 | - |  |  |

**Table S5.** Comparison of the wording of the items and their response options for the International Classification of Functioning, Disability and Health categories of the functioning components common to three of the data sources, using the Model Disability Survey as a reference. (Overview of available functioning data in Switzerland: supporting the use of functioning as a health indicator alongside mortality and morbidity, Switzerland, 2022-2024)

| **ICF code** | |  | | | **Categorization of response option** |
| --- | --- | --- | --- | --- | --- |
| **Data source** | **Item identifier and wording** | **Response option** | | |  |
| **b140 Attention functions** | | | | |  |
| **SHARE** | *MH014_ConcEnter* How is your concentration? For example, can you concentrate on a television programme, film or radio programme? | Difficulty in concentrating on entertainment;  No such difficulty mentioned | | | Confirmation |
| **SHS** | ^†^ *Q51* During the last 2 weeks, how often have you felt affected by the following complaints: Please tick the appropriate answer for each line! (Please select the appropriate answer for each line!)  g) Difficulty concentrating on anything, such as reading the newspaper or watching television? | Never; Several days; More than half the days; Almost every day | | | Frequency |
|  | *1650 ﻿*Do you have difficulty concentrating for more than 10 minutes? | ﻿No, no difficulties; Yes, slight difficulties; Yes, great difficulties; Can't concentrate at all | | | Intensity |
| **Lc65+** | *5.* Have you been bothered, for at least 6 months, by... | Difficulty concentrating on what you are doing. | | | Confirmation |
|  | * ^†^ *23.* Here is a list of sentences describing difficulties that everyone may encounter in daily life. How often do you encounter these difficulties?  c) When I am interrupted in a reading, I have difficulty finding my way back to where I was | Never; Rarely; Sometimes; Often; Very often | | | Frequency |
| **b455 Exercise tolerance functions**** | | | | | |
| **SHS** | ^†^ *Q21.00* Tell me if you have had [the following complaint] over the past 4 weeks, a little or a lot. (If currently taking a medicine for a complaint, it means you have that complaint).  2) General weakness, fatigue, lack of energy. | | Not at all; A little; Strongly | Intensity | |
|  | ^†^ *S6400* How have you felt in the last 4 weeks?  a) Full of life  b) Full of energy  c) Exhausted  d) Tired | Always; Mostly; Sometimes; Rarely; Never | | | Frequency |
| **Lc65+** | *2E a.* For moderate physical efforts such as moving a table, vacuuming, playing bowls, does your health limit you a lot, a little, or not at all? | yes, a lot; yes, a little; no, not at all | | | Intensity |
| **SHP** | *117.* During the last 4 weeks, have you suffered from any of the following disorders or health problems?  General weakness, weariness, or lack of energy | Not at all; Somewhat; Very much. | | | Intensity |
| **d3 Communication** | | | | |  |
| **SHARE** | ^†^ *PH049_HeADLb* Please look at card 12. Please tell me if you have any difficulty with these activities because of a physical, mental, emotional or memory problem. Again exclude any difficulties you expect to last less than three months. | 10. Making telephone calls | | | Confirmation |
|  | *HC889_HealthLiteracy* How often do you need to have someone help you when you read instructions, pamphlets, or other written material from your doctor or pharmacy? | Always; Often; Sometimes; Rarely; Never | | | Frequency |
| **SHS** | *Q14.10 ﻿*Can you follow an ordinary conversation where 2 other people participate? | Yes, without difficulties; Yes, with slight difficulties; Yes, but with strong difficulties; No | | | Intensity |
|  | *Q15.00 ﻿*Can you talk (speak) without difficulty? | Yes, without difficulties; Yes, with slight difficulties; Yes, but with strong difficulties; No | | | Intensity |
|  | ^†^ *Q18.10* I am now going to read you other everyday activities. Please tell me each time whether you can do it without difficulty, with slight difficulty, with strong difficulty, or not at all. b) Make phone calls independently | Yes, without difficulties; Yes, with slight difficulties; Yes, but with strong difficulties; No | | | Intensity |
| **Lc65+** | ^†^ *35.* Do you have difficulty, or do you generally receive help, with the following activities because of physical, mental, emotional or memory problems?  k) Making phone calls | No difficulties; Difficulties, but no help;  I get help | | | Intensity |
| **d450 Walking \| d4500 Walking short distances** | | | | |  |
| **SHARE** | ^†^ *PH048_HeADLa* Please look at card 11. Please tell me whether you have any difficulty doing each of the everyday activities on this card. Exclude any difficulties that you expect to last less than three months. | 1. Walking 100 metres | | | Confirmation |
| **SHS** | *Q16.00* How far can you walk alone i.e. without assistance, without having to stop and without experiencing severe discomfort? | 200M or more;  More than some steps but less than 200M;  Only some steps;  Can't walk at all. | | | Intensity |
| **Lc65+** | ^†^ *30.* Do you, for health reasons, have difficulty in...  a) Walk 100 meters | No difficulty; Some difficulties; Many difficulties or impossible | | | Intensity |
|  | ^†^ *35.* Do you have difficulty, or do you generally receive help, with the following activities because of physical, mental, emotional or memory problems?  b) Walking across a room | No difficulties; Difficulties, but no help;  I get help | | | Intensity |
| **d5 Self-care \| d530 Toileting \| d550 Eating** | | | | |  |
| **SHARE** | *PH049_HeADLb* Please look at card 12. Please tell me if you have any difficulty with these activities because of a physical, mental, emotional or memory problem. Again exclude any difficulties you expect to last less than three months. | 1. Dressing, including putting on shoes and socks  3. Bathing or showering  4. Eating, such as cutting up your food  6. Using the toilet, including getting up or down  11. Taking medication | | | Confirmation |
| **SHS** | ^†^ *Q18.00* (Only to persons 65 years and older. For persons under 65 and persons with disabilities, go to question 18.20). I am now going to read you various activities of daily living. Please tell me each time whether you can do it without difficulty, with slight difficulty, with severe difficulty, or not at all INT: do not include temporary health problems!  a) Eat independently  c) Dressing and undressing independently  d) Going to the toilet independently  e) Bath or shower independently | Yes, without difficulties;  Yes, with slight difficulties;  Yes, but with strong difficulties;  No. | | | Intensity |
| **Lc65+** | ^†^ *35.* Do you have difficulty, or do you generally receive help, with the following activities because of physical, mental, emotional or memory problems?  a) Dressing, including putting on socks and shoes  c) Taking a bath or shower  d) Eating, including cutting food  f) Using the toilet, including sitting down, standing up  l) Preparing and taking medication | No difficulties; Difficulties, but no help;  I get help | | | Intensity |
|  | *52E.* ﻿Do you have any health problems, medications, or anything else that affects the way you eat? | Yes; No | | | Confirmation |
| **d640 Doing housework** | | | | |  |
| **SHARE** | *PH049_HeADLb* Please look at card 12. Please tell me if you have any difficulty with these activities because of a physical, mental, emotional or memory problem. Again exclude any difficulties you expect to last less than three months. | 12. Doing work around the house or garden  15. Doing personal laundry | | | Confirmation |
| **SHS** | ^†^ *Q18.10* I am now going to read you other everyday activities. Please tell me each time whether you can do it without difficulty, with slight difficulty, with strong difficulty, or not at all.  e) Independently do light housework  f) Independently do occasional heavy housework  d) Do laundry independently | Yes, without difficulties;  Yes, with slight difficulties;  Yes, but with strong difficulties;  No. | | | Intensity |
| **Lc65+** | ^†^ *35.* Do you have difficulty, or do you generally receive help, with the following activities because of physical, mental, emotional or memory problems?  g) Doing minor household chores  h) Doing heavy housework | No difficulties; Difficulties, but no help;  I get help | | | Intensity |
| **d860 Basic economic transactions** | | | | |  |
| **SHARE** | *PH049_HeADLb* Please look at card 12. Please tell me if you have any difficulty with these activities because of a physical, mental, emotional or memory problem. Again exclude any difficulties you expect to last less than three months. | 13. Managing money, such as paying bills and keeping track of expenses. | | | Confirmation |
| **SHS** | ^†^ *Q18.10* I am now going to read you other everyday activities. Please tell me each time whether you can do it without difficulty, with slight difficulty, with strong difficulty, or not at all.  g) Take care of finances independently | Yes, without difficulties;  Yes, with slight difficulties;  Yes, but with strong difficulties;  No. | | | Intensity |
| **Lc65+** | ^†^ *35.* Do you have difficulty, or do you generally receive help, with the following activities because of physical, mental, emotional or memory problems?  m) Taking care of money matters, such as paying bills, keeping track of expenses | No difficulties; Difficulties, but no help;  I get help | | | Intensity |

Notes: More than one item can be included in each category, and one item can be coded with several ICF categories, depending on the number of concepts and response options. Higher level categories include all items coded to more detailed levels.

* items with an ICF code of a more detailed level included in a less detailed category. ^†^Only relevant response options are shown.

** Only category common to SHS, Lc65+, and SHP in this comparison.

ICF: International Classification of Functioning, Disability and Health; MDS: Model Disability Survey; SHARE: Survey of Health, Ageing and Retirement in Europe; SHS: Swiss Health Survey; Lc65+: Lausanne cohort 65+; SHP: Swiss Household Panel
